# Myeloperoxidase is not a good biomarker for preeclampsia prediction

**DOI:** 10.1038/s41598-017-09272-4

**Published:** 2017-08-31

**Authors:** L. Rocha-Penha, H. Bettiol, M. A. Barbieri, V. C. Cardoso, R. C. Cavalli, V. C. Sandrim

**Affiliations:** 1 Department of Pharmacology, Institute of Biosciences of Botucatu, Universidade Estadual Paulista (UNESP), Distrito Rubiao Junior, Botucatu, São Paulo 18680-000 Brazil; 2 Department of Pediatrics, Faculty of Medicine of Ribeirao Preto, University of Sao Paulo, Ribeirao Preto, Sao Paulo 14049-900 Brazil; 3 Department of Gynecology and Obstetrics, Faculty of Medicine of Ribeirao Preto, University of Sao Paulo, Ribeirao Preto, São Paulo 14049-900 Brazil

## Abstract

Myeloperoxidase is a proinflammatory enzyme found to be increased in patients with established preeclampsia but never investigated before the disease onset. Here we examined myeloperoxidase concentration and activity in plasma and urine samples from pregnant women who remained normotensive throughout pregnancy and those who developed preeclampsia in order to assess its potential to predict this disorder. Our sample consisted of 30 cases who developed preeclampsia (14 severe and 16 mild) and 57 controls who remained healthy throughout pregnancy, derived from the Brazilian Ribeirão Preto and São Luís prenatal cohort (BRISA). Myeloperoxidase concentration were assessed using a commercial ELISA kit and enzymatic activity through tetramethylbenzidine oxidation. No statistical differences were found in myeloperoxidase levels nor activity between plasma or urine samples from controls, severe and mild cases. Myeloperoxidase did not seem to have a potential application for preeclampsia prediction.

## Introduction

Preeclampsia is a pregnancy-related disorder characterized by new-onset hypertension in association with proteinuria or other systemic signs, and is consider one of the major contributing factors for maternal and fetal morbidity^1^. Although some maternal characteristics are associated with increased risk for development of the disorder^2^, there is still no marker in clinical use for early diagnosis of this syndrome^3,4^.

Complexity of the disorder may be one reason for the difficulty in finding precise biomarkers. Although not fully understood, preeclampsia pathophysiology is mainly characterized in two stages starting with impaired placentation, which leads to ischemia, oxidative stress and release of bioactive factors in maternal circulation, and progressing to the second stage of maternal response when the symptoms arise^5,6^. Several molecules have been investigated for preeclampsia prediction, and the most common are molecules related to angiogenesis. Higher levels of soluble fms-like tyrosine kinase 1 (sFlt-1) and soluble endoglin (sENG), as well as lower levels of placental growth factor (PlGF) were demonstrated before and after preeclampsia onset^6,7^, making of these molecules good candidates for the early diagnosis of this syndrome. However, despite the several promising candidates, their clinical validity is still to be stablished. Although urine samples are advantageous due to its easy, fast and non-invasive collection, and larger volume, research of biomarkers has still been largely focused in plasma/serum samples, thus, the amount of studies related to predictive markers in urine are still scarce.

Maternal systemic inflammation and endothelial dysfunction are consider hallmarks of preeclampsia^8,9^ and increased adhesion molecules^10^ as well as proinflammatory cytokines^6^ were reported in these patients, what may contribute to leukocyte activation and affect endothelial function. A pro-inflammatory molecule able to trigger endothelial dysfunction is the enzyme myeloperoxidase (MPO)^13^, which can impair the vasodilator nitric oxide^12^, reduced in preeclampsia^11^, and generate reactive species contributing for unleashing and perpetuation of the endothelial dysfunction and hypertensive state^13^. Moreover, studies pointed MPO as a possible biomarker for some cardiovascular conditions such as coronary artery disease^12^ and atherosclerosis^14^, but although higher levels of MPO has been demonstrated during preeclampsia^15–18^, there is still no investigation of this enzyme before the disease onset and its predictive potential for this disorder.

Therefore, this study aimed to explore MPO concentration and activity in both plasma and urine samples from pregnant women before preeclampsia onset, in order to verify if this enzyme is a potential biomarker for preeclampsia diagnosis.

## Results

General characteristics for controls who remained healthy throughout pregnancy and for cases who later developed mild or severe preeclampsia are summarized in Table 1. No differences in maternal age, smoke, body mass index and gestational age at sampling were observed. Systolic blood pressure was increased in the severe case group and diastolic blood pressure were also increased in both severe and mild case groups when compared with control group. Besides, severe cases presented lower newborn weight and a higher percentage of small for gestational age infants compared to controls, this group also had lower gestational age at delivery when compared to both controls and mild cases.

**Table 1:** General characteristics of patients enrolled in the study.

Parameters	Healthy pregnant (control)	Preeclampsia (case)		*P*
		Mild	Severe	
*n*	*57*	*16*	*14*	*—*
Age (years)	25.6 ± 6.2	27.1 ± 5.6	29.0 ± 5.8	0.16
Current smoker	5 (8.8)	3 (18.7)	2 (14.3)	0.51
BMI (Kg/m^2^)	29.4 ± 5.8	29.4 ± 5.0	28.3 ± 5.3	0.80
SBP (mmHg)	109.1 ± 10.5	114.8 ± 9.8	119.3 ± 13.4*	0.005
DBP (mmHg)	66.0 ± 6.6	71.9 ± 6.5*	75.9 ± 10.8*	<0.000
GA at sampling (weeks)	23.1 ± 1.2	23.6 ± 1.4	23.1 ± 1.1	0.43
GA at delivery (weeks)	39.7 ± 1.7	38.8 ± 1.1	35.1 ± 4.3*^#^	<0.000
Nulliparous	22 (38.6)	7 (43.7)	4 (28.6)	0.68
NBW (g)	3343.0 ± 495.1	3370.0 ± 520.9	2364.0 ± 1011.0*^#^	<0.000
SGA (%)	2 (3.6)	2 (12.5)	6 (42.9)*	<0.000

Information of this table is referent to the moment of blood and urine collection before preeclampsia diagnosis, except for GA at delivery, NBW and SGA. Data as mean ± SD or n (percentage of total). BMI, body mass index; SBP, systolic blood pressure; DBP, diastolic blood pressure; PE, preeclampsia; NA, non-applicable; GA, gestational age; NBW, newborn weight; SGA, small for gestational weight. ^*^vs healthy pregnant; ^#^vs mild preeclampsia.

**Figure 1:**
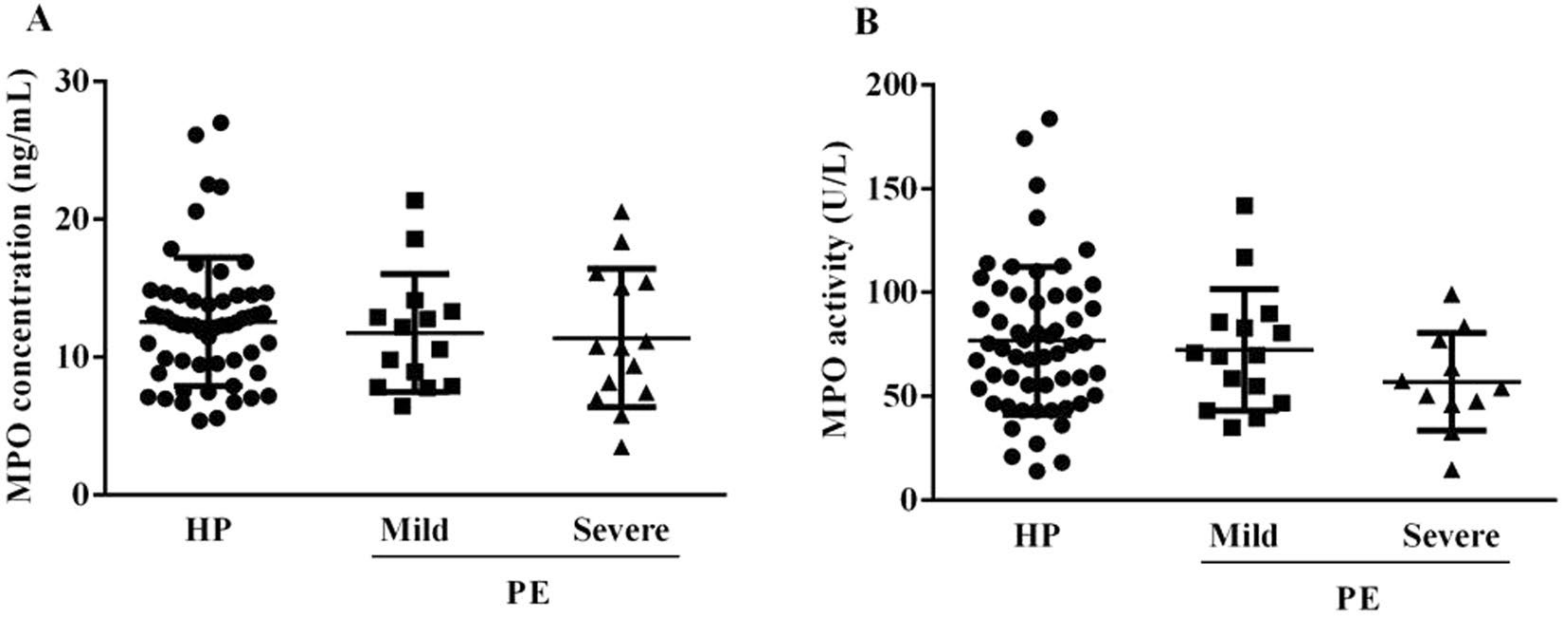
Plasma MPO concentration (**A**) and activity (**B**) from pregnant women who subsequently developed mild (n = 14) and severe (n = 14) preeclampsia (PE), and those that remained normotensive throughout pregnancy (HP; n = 56). No significant difference on MPO levels (*P* = 0.6) nor activity (*P* = 0.2) between HP (12.5 ± 4.7 ng/mL and 76.7 ± 35.6 U/L), mild PE (11.7 ± 4.3 ng/mL and 72.3 ± 29.3 U/L) and severe PE (11.4 ± 5.0 ng/mL and 57.0 ± 23.6 U/L) respectively. Data as mean ± SD. Comparison between groups were by ANOVA 1-way followed by Tukey’s multiple comparisons test.

While investigating plasma MPO concentration (Fig. 1A) and activity (Fig. 1B) no statistical differences were observed among subjects who remained normotensive throughout pregnancy (controls) and those who developed any of the two forms of preeclampsia (mild and severe cases) (*P* = 0.6 and *P* = 0.2, respectively). However, despite no statistical significance, it is possible to observe a slight decrease in enzymatic activity in the group of severe cases when compared to controls (*P* = 0.2; 57.0 ± 23.6 *vs* 76.7 ± 35.6 U/L, respectively).

**Figure 2:**
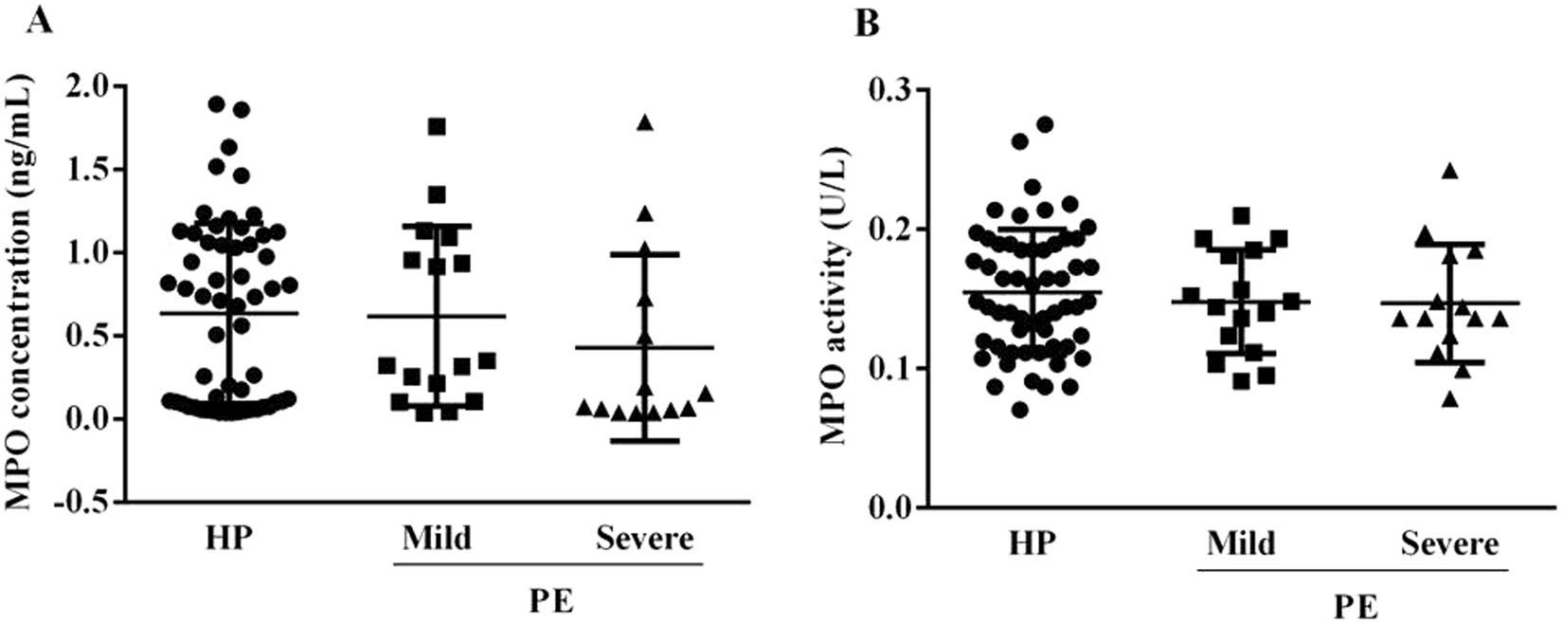
Urine MPO concentration (**A**) and activity (**B**) from pregnant women who subsequently developed mild (n = 16) and severe (n = 14) preeclampsia (PE), and those that remained normotensive throughout pregnancy (HP; n = 57). No significant differences on MPO levels (*P* = 0.2) nor activity (*P* = 0.7) between HP (0.7 [0.04–0.9] ng/mL and 0.1 ± 0.05 U/L), mild PE (0.3 [0.04–1.7] ng/mL and 0.1 ± 0.04 U/L) and severe PE (0.1 [0.04–1.8] ng/mL and 0.1 ± 0.04 U/L), respectively. Data as median [minimum–maximum] or mean ± SD. Comparison of concentration between groups were by Kruskal-Wallis followed by Dunn’s or ANOVA 1-way followed by Tukey’s multiple comparisons test.

Regarding MPO measurements in urine samples, similarly to plasma, we did not found significant differences in enzyme concentration (Fig. 2A) nor activity (Fig. 2B) between controls and mild/severe cases (*P* = 0.2 and *P* = 0.7, respectively). Nevertheless, there is a slight decrease in MPO concentration in severe case group compared to control (*P* = 0.2; 0.4 ± 0.5 *vs* 0.6 ± 0.5 ng/mL, respectively).

In order to investigate some possible association, we correlated MPO concentration and activity in both plasma and urine samples between itself, with maternal age, body mass index, systolic and diastolic blood pressure, gestational age at sampling, newborn weight (Table 2) and smoking status. Despite no statistical difference in body mass index among the three groups, a positive correlation (*P* = 0.02; r = 0.6) between body mass index and plasma MPO concentration in severe case group was found (Supplementary Figure S1A). Also among severe cases, a negative correlation (*P* = 0.01; r = −0.7) was observed between plasma MPO activity and systolic blood pressure (Supplementary Figure S1B). In control group, it was found a significant positive correlation (*P* = 0.01; r = 0.4) between MPO concentration and activity in urine samples (Supplementary Figure S1C). No other statistically significant correlations were found. Concerning to the differences in MPO related to smoking status, this characteristic do not seems to influence in MPO concentration nor activity (P ≥ 0.2 and P ≥ 0.1, respectively; data not shown).

**Table 2:** Correlations between general characteristics and MPO concentration and activity in plasma and urine samples from controls, mild and severe cases

Parameters	Healthy pregnant (controls)				Mild preeclampsia (case)				Severe preeclampsia (case)			
	Plasma		Urine		Plasma		Urine		Plasma		Urine	
	Conc.	Activity	Conc.	Activity	Conc.	Activity	Conc.	Activity	Conc.	Activity	Conc.	Activity
SBP (mmHg)	0.4(−0.1)	0.5(0.1)	0.4(−0.1)	0.6(−0.1)	0.3(−0.3)	0.6(−0.1)	1.0(−0.0)	0.6(0.1)	0.5(0.2)	**0.01(−0.7)**	0.3(−0.3)	0.9(0.0)
DBP (mmHg)	0.7(−0.0)	0.5(0.1)	0.7(−0.0)	1.0(−0.0)	0.1(−0.4)	0.1(−0.4)	0.7(−0.1)	0.5(−0.2)	0.6(0.1)	0.1(−0.6)	0.1(−0.5)	0.3(0.3)
BMI (Kg/m^2^)	0.4(0.1)	0.5(0.1)	0.5(−0.1)	0.7(−0.0)	0.2(−0.4)	0.2(−0.3)	0.2(−0.3)	0.5(−0.2)	**0.02(0.6)**	0.6(0.2)	0.4(−0.2)	0.3(0.3)
Maternal age (years)	0.5(−0.1)	0.7(0.1)	0.7(0.0)	1.0(−0.0)	0.8(−0.1)	0.1(−0.5)	0.8(−0.1)	0.7(0.1)	0.4(−0.3)	0.6(0.2)	0.7(−0.1)	0.8(−0.1)
GA at sampling (weeks)	0.6(−0.1)	0.7(0.1)	0.7(−0.0)	0.7(−0.0)	0.5(−0.2)	0.7(0.1)	0.6(−0.1)	0.4(−0.2)	0.6(0.2)	0.7(0.1)	1.0(−0.0)	0.9(−0.0)
NBW (g)	0.5(−0.1)	0.8(−0.0)	0.5(−0.1)	0.5(0.1)	0.1(−0.5)	0.9(0.0)	0.8(0.1)	0.8(0.1)	0.7(−0.1)	0.4(0.3)	0.4(0.3)	0.3(0.3)
MPO levels × activity	0.3 (0.1)		**0.01(0.4)**		0.5(−0.2)		0.6(0.1)		0.5(0.2)		0.5(−0.2)	

Data as *P* value (correlation coefficient).; SBP, systolic blood pressure; DBP, diastolic blood pressure; BMI, body mass index GA, gestational age; NBW, newborn weight. Statistically significant (*P* ≤ 0.05).

## Discussion

In this study, we aimed to explore MPO concentration and activity in both plasma and urine samples from pregnant women who remained normotensive throughout pregnancy and those who subsequently developed preeclampsia in order to verify if this molecule could be useful for early diagnosis of this disease. To our knowledge, we are the first to investigate the potential of MPO for preeclampsia prediction. However, plasmatic as well as urinary MPO concentration and activity showed no significant difference between healthy controls and pregnant women who later developed mild or severe preeclampsia.

Previous studies have investigated MPO in established preeclampsia, some reporting higher concentration^15,16^ and activity^17,18^ in plasma/serum samples from preeclamptic patients compared to healthy pregnant women, and others pointing no differences^19–21^. Although urine samples are able to provide important information about several conditions of the organism with a fast, simple and non-invasive collection, no previous study has investigated MPO parameters in this matrix from preeclamptic patients. Besides, none has explored a possible contribution of this enzyme for early diagnosis of this disorder.

In both sample matrix, we did not found any difference in enzyme levels or activity when comparing controls, mild and severe cases, similarly to Hung *et al*.^19^, who also observed no differences in plasma MPO levels at 15-20 weeks gestation between healthy pregnant women and women who developed preeclampsia, thus suggesting that the increase in MPO observed by others^15,16^ after preeclampsia diagnosis may be a consequence instead of a trigger for the disorder. Although no statistical differences has been found in MPO parameters between control and cases, a decreasing trend can be observed in urine MPO concentration and in plasma MPO activity on patients with severe preeclampsia. This decreasing trend might be related to the severity of the disease itself; higher blood pressure as well as other complications^1^ of the severe form of the disease may contribute to the oxidative state, resulting in increased levels of reactive oxygen species as superoxide and hydrogen peroxide. These reactive species when in high levels are able to inactivate MPO by converting the molecule to an inactive form, and to change the enzyme structure by destructing the heme-groups and iron release^13,22^. Moreover, ceruloplasmin levels, increased in preeclampsia^23^, as well as some polyphenols derived from diet can affect MPO by reacting directly with the enzyme, thus decreasing its activity, or by reacting with its end products^24,25^.

A possible explanation for the lack of difference in MPO parameters between the groups when comparing to other studies is the gestational age at sampling. Previous studies^15–18^ that reported some differences in MPO between preeclampsia and healthy pregnant women evaluated samples collected with a gestational age above 30 weeks, while our samples were collected about 23 weeks of gestation, what might indicate that the increased MPO in preeclampsia pointed by others may not be a causative factor but a result of the established disease. It is known that, by the end of second trimester, poor trophoblast invasion and consequently impaired remodeling of the uterine spiral arteries result in a placental ischemia with a subsequent release of bioactive factors in maternal circulation of preeclampsia patients. Some of these factors, mostly proinflammatory cytokines as TNFα^26^, as well as the hypoxic condition^27^ itself could stimulate activation of neutrophils and release of MPO from those cells. Moreover, Hung *et al*.^28^ demonstrated an increase in oxidative stress on healthy pregnant women on the third trimester (above 26 weeks of gestation) which could also improve MPO activity due to the higher availability of co-factors as superoxide and hydrogen peroxide.

Analyzing correlations of MPO with other parameters, we observed a positive correlation in severe case group between plasma MPO concentration and body mass index. Among other maternal characteristics, obesity is consider a risk factor for the development of preeclampsia as demonstrated by Bodnar and cols^29,30^, which observed that elevated body mass index increased the incidence of both mild and severe forms of this disorder. In the past years, obesity cases have been increasing considerably, and one of its results is the chronic inflammatory status triggered by the metabolic excess and adipose tissue activation, which leads to production and release of substances able to modulate the inflammatory and immune response^31^. This condition contribute to macrophage and neutrophil activation and consequent release of MPO, which was demonstrated to be increased in obese subjects in the study of Borato *et al*.^32^. Moreover, Shukla *et al*.^33^ demonstrated greater MPO staining in systemic vasculature from both obese and preeclamptic women, suggesting that overweight women could display higher risk for developing this disorder. There was also a negative correlation in severe case group between plasma MPO concentration and systolic blood pressure that, although unexpected, might suggest an attempt by the organism to normalize the blood pressure, already increased, in the presence of a molecule which contribute to vasoconstriction (mostly through nitric oxide consumption).

Some authors also investigated MPO levels in placental samples, but the results obtained were contradictory. Gandley *et al*.^16^ observed higher levels of the enzyme in placental extracts from patients with preeclampsia compared with normal pregnant women, while Hung *et al*.^19^ reported no differences between the groups. One of the reasons for this difference between authors might be the placental sampling site; in the work of Gandley *et al*.^16^ MPO was quantified in a single site of placenta, while Hung *et al*.^19^ measured the enzyme in a pool of different sites of the organ, what might influence the final levels of MPO.

Although we were the first to investigate MPO predictive potential in preeclampsia by measuring both concentration and activity in two different matrices (plasma and urine), this study present some limitations. As a convenience cohort, the BRISA cohort was finished once sample number was reached, and this cohort does not restrict patients with previous diseases. Moreover, we did not evaluated MPO parameters in placental samples, so we do not know the enzyme levels nor activity in this organ among our subjects. Another limitation might be related to the method applied for MPO activity that, although still one of the most common applied so far^34^, due to the lack of a standard method for plasma/urine samples might present some interferences generally in the presence of other peroxidases. However, we have demonstrated^35^ that most of the enzymatic activity assessed in plasma samples using the colorimetric method with TMB (tetramethylbenzidine) as substrate is mainly referent to MPO activity, once we observed that enzymatic activity detection decreased in more than 90% after addition of 4-aminobenzoic acid hydrazide, a MPO specific and irreversible inhibitor. Therefore, although susceptible to some interferences, it seems to be a reasonable method for assessment of MPO activity.

In conclusion, this study did not found any evidence that MPO, concentration or activity, was elevated before the diagnosis of preeclampsia, probably because the MPO increase might happen as a result from proinflammatory stimulus after the establishment of the disease. However, the correlation between body mass index and MPO levels suggest an increased risk for the development of this disorder. Therefore, in view of the results, MPO does not seem to display a potential use as a marker for preeclampsia prediction.

## Material and Methods

### Subjects

This predictive study is part of a more broad observational study accomplished in two Brazilian cities (BRISA)^36^. A total of 1,417 pregnant women were evaluated at *Hospital das Clínicas of Ribeirão Preto*–University of São Paulo at 20-25 weeks of gestation (Prenatal cohort), from these 17 did not return and 460 gave birth in other units outside the institution. From the 940 pregnant remaining, 30 developed preeclampsia (cases) and 58 healthy pregnant women were draw from the remaining healthy pregnant group; subjects’ selection is outlined in Supplementary Figure S2. Preeclampsia was defined following guidelines of NHBPEP^37^ (National High Blood Pressure Education Program Working Group on High Blood Pressure in Pregnancy). Severity of the disorder was defined according to the American College of Obstetricians and Gynecologists^1^, and preeclampsia was classified as severe when systolic blood pressure $\ge$160 mmHg and/or diastolic blood pressure $\ge$110 mmHg or in the presence of manifestations as thrombocytopenia, impaired liver function, progressive renal insufficiency, pulmonary edema and cerebral or visual disturbances. All participants provided written informed consent, and the study was approved by Institutional Review Board of the University of Sao Paulo, Ribeirão Preto (reference 4116/2008), according to the declaration of Helsinki. Maternal venous blood samples were collected in *Vacutainer* tubes (Becton-Dickinson, São Paulo, Brazil) with EDTA as anticoagulant. Samples were centrifuged at room temperature and plasma were stored at −80 °C until analysis.

### Myeloperoxidase concentration and activity

MPO concentration was evaluated in plasma/urine samples using a commercial ELISA kit (Human Myeloperoxidase DuoSet ELISA - R&D Systems, Minneapolis, MN, USA) with standard range between 62.5–4,000.0 pg/mL. Plasma, but not urine, samples were diluted 1:100 in reagent diluent (1% BSA in PBS) and optical density was determined at 450 nm using in a microplate reader (Synergy 4–BioTek, Winooski, VT, USA). Intra-assay of 1.25% and 1.98% for urine and plasma analysis, respectively.

MPO activity was determined based in the method of Bradley *et al*.^38^ by measuring tetramethylbenzidine (TMB) oxidation in the presence of hydrogen peroxide. Briefly, 30 µL of urine/plasma (plasma diluted 1:100 in PBS) were incubated with 20 µL of PBS and 100 µL of liquid substrate system (Sigma, St. Louis, MO, USA), composed by TMB and hydrogen peroxide, at 37 °C for 10 minutes protected from light. After incubation, the reaction was blocked with 100 µL sulfuric acid 2 N and optical density were determined at 450 nm. A standard curve (range 0.153–2500 mU/50 µL) was generated by incubation of horseradish peroxidase with the previous reagents. Intra-assay of 5.45% and 2.51% for urine and plasma analysis, respectively.

### Statistical analysis

Statistical analysis was performed with GraphPad Prism for Windows, version 6.01 (GraphPad Software, San Diego, CA, USA). Categorical variables were compared by *χ*^2^ tests and continuous variables were compared by Student t test, ANOVA followed by Tukey test (for normally distributed variables) or Mann-Withney’s test, Kruskal-Wallis followed by Dunn’s multiple comparison test (for not normally distributed variables). Correlations were analyzed using Pearson’s test, except for MPO levels x activity that were analyzed by Spearman’s test. For all tests a probability value of *P* < 0.05 was considered significant.

## Electronic supplementary material

SUPPLEMENTARY INFO
