# Author Correction: Myeloperoxidase is not a good biomarker for preeclampsia prediction

**DOI:** 10.1038/s41598-018-22636-8

**Published:** 2018-03-08

**Authors:** L. Rocha-Penha, H. Bettiol, M. A. Barbieri, V. C. Cardoso, R. C. Cavalli, V. C. Sandrim

**Affiliations:** 1 Department of Pharmacology, Institute of Biosciences of Botucatu, Universidade Estadual Paulista (UNESP), Distrito Rubiao Junior, Botucatu, São Paulo 18680-000 Brazil; 2 Department of Pediatrics, Faculty of Medicine of Ribeirao Preto, University of Sao Paulo, Ribeirao Preto, Sao Paulo 14049-900 Brazil; 3 Department of Gynecology and Obstetrics, Faculty of Medicine of Ribeirao Preto, University of Sao Paulo, Ribeirao Preto, São Paulo 14049-900 Brazil

Correction to: *Scientific Reports* 10.1038/s41598-017-09272-4, published online 31 August 2017

The Acknowledgements section in this Article is incomplete.

“The authors acknowledge the Conselho Nacional de Desenvolvimento Científico e Tecnológico (CNPq-Brazil), the Coordenação de Aperfeiçoamento de Pessoal de Nível Superior (CAPES-Brazil), and the Fundação de Amparo à Pesquisa do Estado de São Paulo (FAPESP-Brazil) for financial help, as well as the team involved in BRISA study.”

should read:

“The authors acknowledge the Conselho Nacional de Desenvolvimento Científico e Tecnológico (CNPq-Brazil; grant #2014-5/305587), the Coordenação de Aperfeiçoamento de Pessoal de Nível Superior (CAPES-Brazil), and the Fundação de Amparo à Pesquisa do Estado de São Paulo (FAPESP-Brazil grants #2017/14546-6, #2015/20461-8 and #2008/53593-0) for financial help, as well as the team involved in BRISA study.”

