# Supplementary material for: Myeloperoxidase is not a good biomarker for preeclampsia prediction
Source: Sci Rep. 2017 Aug 31;7:10257. doi: 10.1038/s41598-017-09272-4 (PMC5579011; doi:10.1038/s41598-017-09272-4)
Supplement: Supplementary file 1 — SUPPLEMENTARY INFO [file 41598_2017_9272_MOESM1_ESM.doc]

Supplementary Information

**Myeloperoxidase is not a good biomarker for preeclampsia prediction**

Authors’ names and affiliations:

Rocha-Penha L ^1^, Bettiol H ^2^, Barbieri MA ^2^, Cardoso VC ^2^, Cavalli RC ^3^, Sandrim VC ^1*^

1-Department of Pharmacology, Institute of Biosciences of Botucatu, Universidade Estadual Paulista (UNESP), Distrito Rubiao Junior, Botucatu, São Paulo, 18680-000, Brazil.

2 - Department of Pediatrics, Faculty of Medicine of Ribeirao Preto, University of Sao Paulo, Ribeirao Preto, Sao Paulo, 14049-900, Brazil.

3- Department of Gynecology and Obstetrics, Faculty of Medicine of Ribeirao Preto, University of Sao Paulo, Ribeirao Preto, São Paulo, 14049-900, Brazil.

**Supplementary Figure S1.** Statistically significant correlations. In severe cases of preeclampsia **(A)** a positive correlation between plasma MPO concentration and BMI (*P*=0.02, r=0.6) and **(B)** a negative correlation between plasma MPO activity and SBP (*P*=0.01, r=-0.7). In controls **(C)** a positive correlation between MPO concentration and activity (*P*=0.01, r=0.4). Correlations assessed by Pearson’s test. MPO, myeloperoxidase; BMI, body mass index; SBP, systolic blood pressure.

**
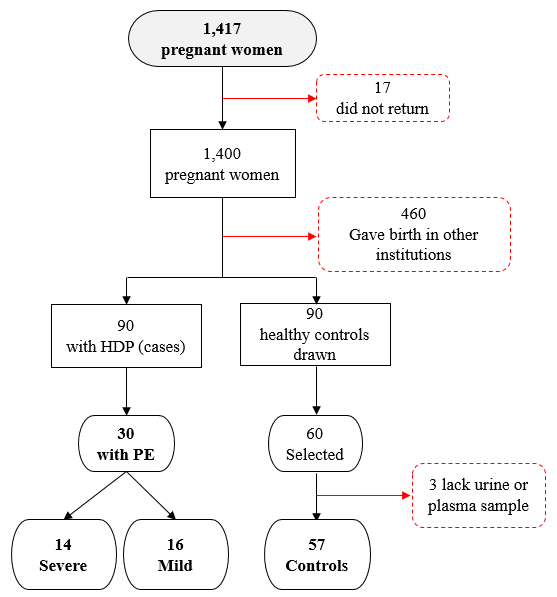
**

**Supplementary Figure S2.** Flowchart of the subject selection. HDP, hypertensive disorders of pregnancy; PE, preeclampsia.
